# Differential selection on gene translation efficiency between the filamentous fungus *Ashbya gossypii *and yeasts

**DOI:** 10.1186/1471-2148-8-343

**Published:** 2008-12-29

**Authors:** Huifeng Jiang, Yue Zhang, Jun Sun, Wen Wang, Zhenglong Gu

**Affiliations:** 1 Division of Nutritional Sciences, Cornell University, Ithaca, NY 14853, USA; 2 CAS-Max Planck Junior Research Group, State Key Laboratory of Genetic Resources and Evolution, Kunming Institute of Zoology, Chinese Academy of Sciences (CAS), Kunming, Yunnan 650223, PR China; 3 Graduate School of Chinese Academy Sciences, Beijing 100039, PR China; 4 State Key Laboratory of Cellular and Molecular Evolution, Kunming Institute of Zoology, Chinese Academy of Sciences, Kunming 650223, Yunnan Province, PR China

## Abstract

**Background:**

The filamentous fungus *Ashbya gossypii *grows into a multicellular mycelium that is distinct from the unicellular morphology of its closely related yeast species. It has been proposed that genes important for cell cycle regulation play central roles for such phenotypic differences. Because *A. gossypii *shares an almost identical set of cell cycle genes with the typical yeast *Saccharomyces cerevisiae*, the differences might occur at the level of orthologous gene regulation. Codon usage patterns were compared to identify orthologous genes with different gene regulation between *A. gossypii *and nine closely related yeast species.

**Results:**

Here we identified 3,151 orthologous genes between *A. gossypii *and nine yeast species. Two groups of genes with significant differences in codon usage (gene translation efficiency) were identified between *A. gossypii *and yeasts. 333 genes (Group I) and 552 genes (Group II) have significantly higher translation efficiency in *A. gossypii *and yeasts, respectively. Functional enrichment and pathway analysis show that Group I genes are significantly enriched with cell cycle functions whereas Group II genes are biased toward metabolic functions.

**Conclusion:**

Because translation efficiency of a gene is closely related to its functional importance, the observed functional distributions of orthologous genes with different translation efficiency might account for phenotypic differentiation between *A. gossypii *and yeast species. The results shed light on the mechanisms for pseudohyphal growth in pathogenic yeast species.

## Background

Filamentous and yeast-like growths are two ways for fungi to propagate in nature. Under normal growth conditions, filamentous fungi grow as multicellular mycelium but yeasts form unicellular colonies. Most species in the clade *Saccharomycotina *are yeasts. In some special environments, these species can switch to pseudohyphal growth that is morphologically similar to filamentous growth, but they can not form real hyphal filaments [1,2]. *Ashbya gossypii*, however, is classified as a filamentous fungus because it forms natural hyphal filaments for most of its life cycle [3,4]. What the nature of the molecular differences between *A. gossypii *and yeasts is that led to such a phenotypic divergence remains an open question. It has been proposed that the divergence might result from a modified cell cycle processes in *A. gossypii *[4-6]. However, comparative genomics studies reveal that *A. gossypii *shares an almost identical set of cell cycle genes with the typical yeast *Saccharomyces cerevisiae *[5]. Although loss or gain of genes among different species often play important roles in phenotypic diversity [7], divergence of regulation in orthologous genes can also contribute significantly to this process [8]. The fact that *A. gossypii *and its closely related yeasts share a very similar set of cell cycle genes prompts us to investigate whether these genes have unique regulation patterns in *A. gossypii*.

Genetic codes exhibit significant usage bias. Selection for gene translation efficiency is the most common Darwinian explanation for codon usage bias. Under this scenario, codons corresponding to abundant tRNA would be preferentially used [9-12]. Natural selection biases such preferred codons so as to enhance accuracy and speed of protein synthesis in highly expressed genes [13-15]. Therefore, differential translation efficiency reflects protein expression difference, which in return can indicate differential functional requirements for these proteins [14]. Therefore, divergence of translation efficiency could reveal orthologous gene functional differentiation across species [15]. By comparing codon usage patterns for orthologous genes between *A. gossypii *and nine yeast species [2], we show in this study that in *A. gossypii *genes with significantly higher translation efficiency are enriched in cell cycle processes, whereas genes with significantly lower translation efficiency are enriched in metabolic processes. Both these findings could account for phenotypic differentiation between multicellular *A. gossypii *and unicellular yeasts, and would shed light on the evolutionary processes that enable pseudohyphal growth in pathogenic yeast species.

## Results

### tRNA adaptation index (tAI)

Within a genome, codon usage, which matches tRNA pool of the organism, is an important indicator for gene translational efficiency [16]. In order to detect the differences in gene translational efficiency between *A. gossypii *and yeasts, a gene matrix containing 3,151 orthologous gene groups for *A. gossypii *and nine yeast species was constructed. Species-specific tRNA adaptation index (tAI), a measurement that evaluates translation efficiency of coding sequences [17], was predicted for each gene based on tRNA pool in that species. Because estimation of codon usage can be affected by species-specific factors such as genome-wide GC contents (Additional file 1), in order to have cross species comparisons, we normalized the tAI value of each gene by the median tAI value of all studied orthologous genes in the same species. The tAI values from nine yeast species were used to calculate the likelihood of observing the tAI value for *A. gossypii *gene in each orthologous gene group.

**Figure 1:**
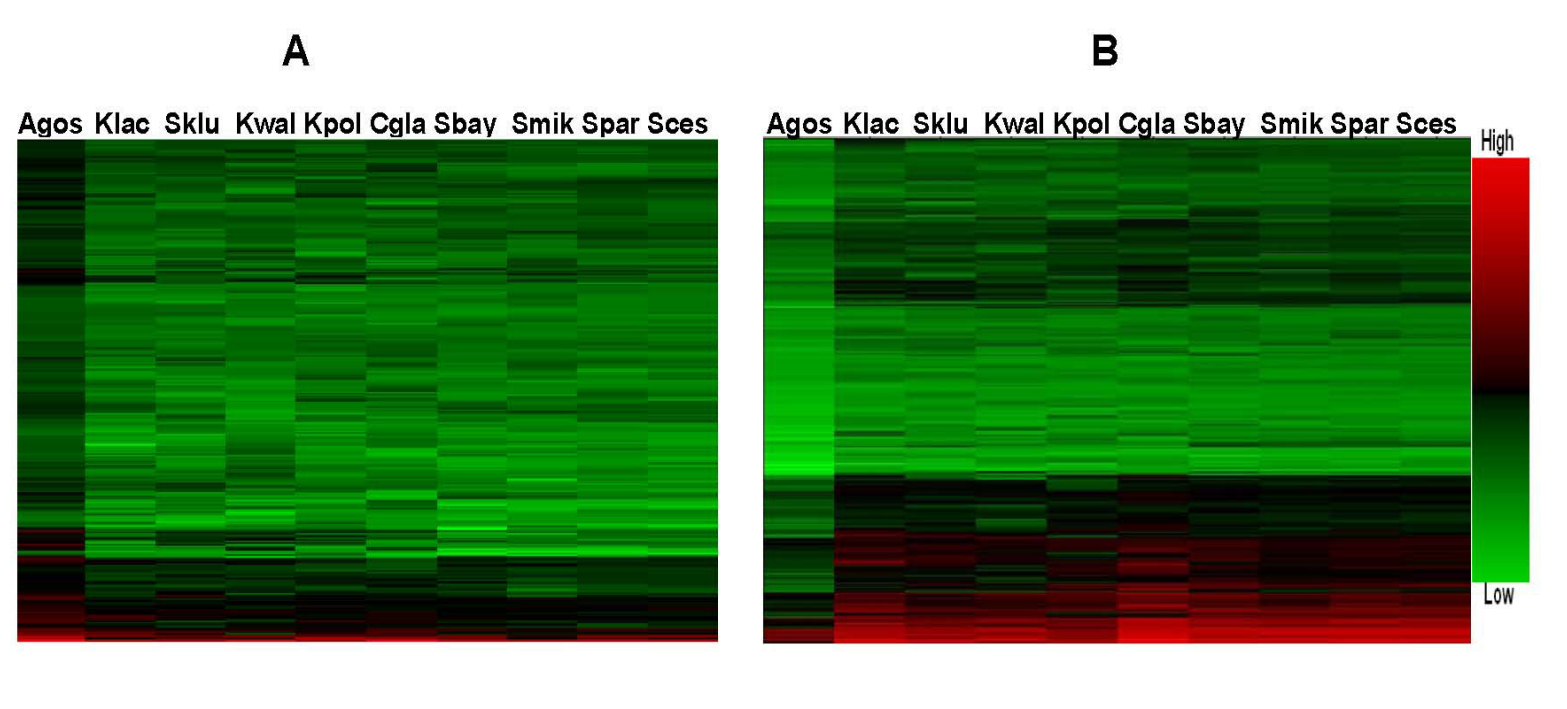
**tAI profile of orthologous gene in Group I and II**. A) tAI values for orthologous genes in Group I (tAI values in *A. gossypii *are significantly higher than those in yeasts); B) tAI values for orthologous genes in Group II (tAI values in *A. gossypii *are significantly lower than those in yeasts). The scale of tAI values is shown to the right of the figure. (Agos: *Ashbya gossypii*; Sces: *Saccharomyces cerevisiae; *Spar:*Saccharomyces paradoxus; *Smik:*Saccharomyces mikatae; *Sbay:*Saccharomyces bayanus; *Cgla:*Candida glabrata; *Kpol:*Kluyveromyces polysporus; *Kwal:*Kluyveromyces waltii*, Sklu: *Saccharomyces kluyveri; *Klac:*Kluyveromyces lactis*).

Based on statistical significance at the 95% confidence level, we divided all orthologous genes into three categories. Group I: 333 genes showing significantly higher tAI values in *A. gossypii *than in yeasts (Figure 1A), group II: 552 genes showing significantly lower tAI values in *A. gossypii *than in yeasts (Figure 1B) and group III: the rest of 2,266 genes that have no obviously different tAI values between *A. gossypii *and yeasts (Additional file 2 for tAI values of genes in Group III). Under the assumption that translation efficiency is a selected trait, genes in group I would have higher expression and be translated more efficiently in *A. gossypii *than their orthologous genes in yeasts, whereas genes in group II would show the opposite pattern.

### GO (Gene Ontology) functional enrichment analysis

**Figure 2:**
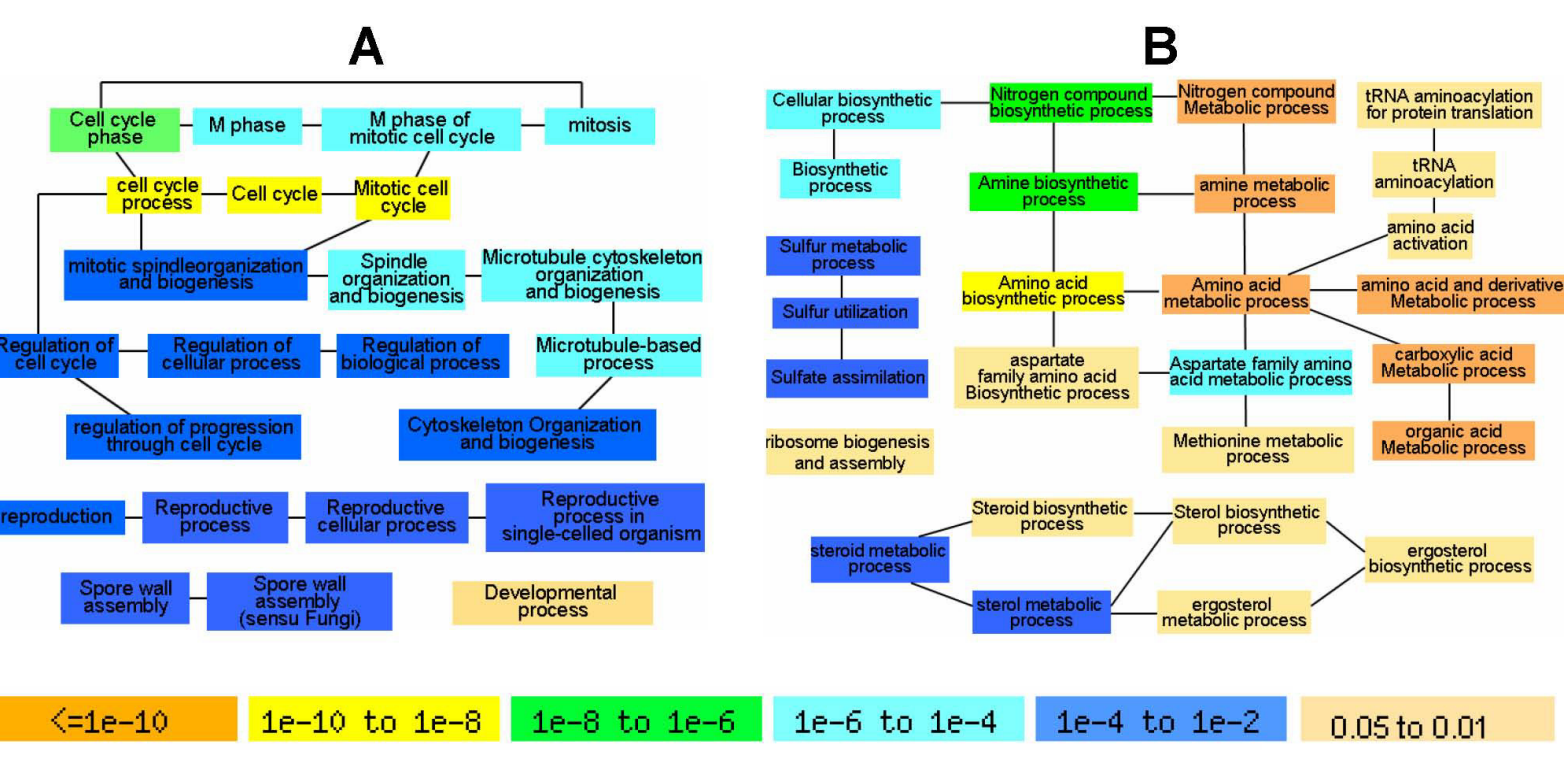
**Distribution of enriched GO functions for genes in Group I and II**. A) Biological functions with enriched gene representation in Group I; B) Biological functions with enriched gene representation in Group II. The edges between functional categories represent their hierarchical relationships in Gene Ontology annotation database. The colour of each box denotes *P*-value from GO term finder software (See additional file 3 for the table list of these functional categories with FDR analysis).

Different tAI values for orthologous genes indicate the distinct functional importance of these genes in compared species [15]. Using GO terms in *S. cerevisiae *as reference, we characterized functional enrichment for Group I and Group II genes, respectively [18]. As shown in Figure 2A (See additional file 3 for the table list of these functional categories), Group I genes display significant enrichment in functions such as cell cycle regulation, mitosis and cytoskeleton organization. Interestingly, these functions have been hypothesized to be important for the unique life style in *A. gossypii *[6]. In addition, Group I genes also show enrichment of functions that are related with reproduction process, spore wall assembly and developmental process. By contrast, Group II genes show functional enrichment in amino acid and steroid biosynthesis (Figure 2B). Different from above two groups, Group III genes do not show any functional enrichment (data not shown).

### KEGG (Kyoto Encyclopedia of Genes and Genomes) pathway analysis

**Figure 3:**
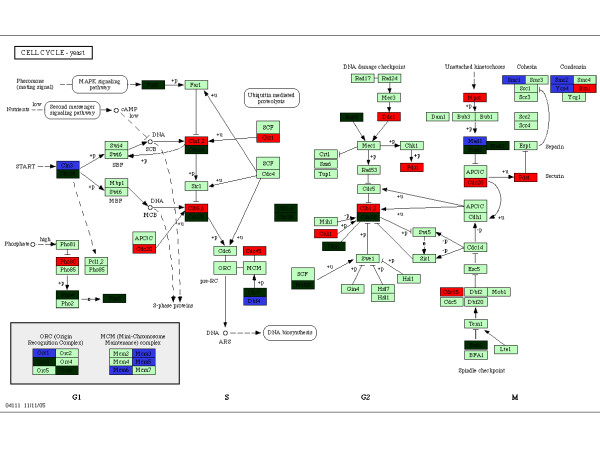
**Cell cycle pathways in *S. cerevisiae***. The pathway figure was downloaded from KEGG database. Genes from Group I, II and III were denoted in red, blue and light green, respectively. Genes with dark green were not included in our orthologous matrix because they might be missing from one of the studied species.

In order to further see the impact of differences in gene translational efficiency, we assigned all studied orthologous genes into identified pathways according to KEGG annotation for *S. cerevisiae *[19]; 897 genes (75% of all annotated genes in KEGG pathways) are included in our orthologous matrix. Among them, 79 genes belong to Group I and 219 genes belong to Group II. A Chi-square test was used to estimate distribution bias for each pathway. With a cut-off *P *value of 0.01, six pathways show significant distribution bias (Table 1). Consistent with gene ontology analysis, two of the three pathways enriched with Group I genes (cell cycle pathway and ubiquitin mediated proteolysis) are related to cell cycle regulation. As shown in Figure 3, cell cycle genes that belong to Group I scatter at each phase of cell division, so the enhanced translation efficiency, hence the increased importance of these genes, would influence the whole cell division process in *A. gossypii*. It is noteworthy that some cell cycle genes also belong to Group II. Interestingly, almost half of them are related with two protein complexes (condensin and mini-chromosome maintenance complex, Figure 3). Group II genes are enriched in metabolic processes such as lysine biosynthesis, steroid biosynthesis and aminoacyl-tRNA biosynthesis. At 95% confidence level another six biosynthesis pathways are enriched with Group II genes (See additional file 4: Table 1 for the details of these pathways).

**Table 1:** KEGG pathways that are enriched in Group I or Group II genes

Pathway Name	KEGG ID	Total gene number	Gene num. in Group I	Gene num. in Group II	Gene num. in Group III	*P*-value
**Cell cycle – yeast **^**a**^	04111	83	19	12	52	<0.01
**Basal transcription factors **^**a**^	03022	23	6	1	16	<0.01
**Ubiquitin mediated proteolysis **^**a**^	04120	20	7	2	11	<0.01
**Lysine biosynthesis **^**b**^	00300	14	0	10	4	<0.01
**Biosynthesis of steroids **^**b**^	00100	16	0	10	6	<0.01
**Aminoacyl-tRNA biosynthesis **^**b**^	00970	34	0	16	18	<0.01

a: Enriched with Group I genes; b: Enriched with Group II genes.

### Comparative analysis with experimentally confirmed genes in *A. gossypii*

The above observations are all based on functional annotations for genes in *S. cerevisiae*. In order to confirm that genes with significantly higher tAI values in *A. gossypii *are truly enriched in cell cycle pathway genes, we identified all studied genes in *A. gossypii *according to NCBI database. Twenty one genes in *A. gossypii *were confirmed to be related with cell cycle processes (Table 2). Eight of them have significantly higher tAI values in *A. gossypii *(Group I, 333 gene), the other 13 genes do not show significant differences of tAI values among species (Group III, 2,266 genes). Fisher's exact test indicates that these confirmed cell cycle genes in *A. gossypii *are also enriched in Group I (*P *= 0.003).

**Table 2:** Experimentally confirmed cell cycle genes in *A. gossypii*

Gene name in *A. gossypii*	Orthologous ORF name in *S. cerevisiae*	***P*-value **^**a**^	References
AgCDC16	YKL022C	0.000243	Gladfelter et al. 2007 [35]
AgCDC42	YLR229C	0.00143	Wendland et al. 2001 [36]
AgCDC24	YAL041W	0.001578	Wendland et al. 2001 [36]
AgCDC20	YGL116W	0.001869	Gladfelter et al. 2007 [35]
AgDOC1	YGL240W	0.004167	Gladfelter et al. 2007 [35]
AgRHO4	YKR055W	0.004777	Wendland et al. 2005 [4]
AgCLB5	YPR120C	0.007449	Hungerbuehler et al. 2007 [37]
AgPDS1	YDR113C	0.048015	Gladfelter et al. 2007 [35]
AgBEM2	YER155C	0.07874	Wendland 2000a [38]
AgSWE1	YJL187C	0.117698	Helfer et al. 2006 [39]
AgBEM3	YPL115C	0.122939	Wendland et al. 2005 [4]
AgCDH1	YGL003C	0.127995	Gladfelter et al. 2007 [35]
AgRSR1	YGR152C	0.148734	Bauer et al. 2004 [40]
AgCDC23	YHR166C	0.198647	Gladfelter et al. 2007 [35]
AgBOI1	YBL085W	0.2807	Knechtle et al. 2006 [41]
AgBNI1	YNL271C	0.281555	Schmitz et al. 2005 [42]
AgSIC1	YLR079W	0.787007	Gladfelter et al. 2006 [20]
AgRHO1	YPR165W	0.800873	Walther et al. 2005 [43]
AgSPA2	YLL021W	0.812361	Knechtle et al. 2003 [44]
AgBUD3	YCL014W	0.821338	Wendland 2003 [45]
AgCYK1	YPL242C	0.90517	Wendland 2002 [46]

a: Comparison of tAI values between *A. gossypii *and other yeast species.

## Discussion

### Codon usage bias in *A. gossypii*

Among studied species, *A. gossypii *has the highest genome-wide GC content (0.53), while the GC contents in other species are from 0.35 to 0.46 (Additional file 1A). Correlation coefficients of codon usage between *A. gossypii *and other nine species show strongly positive relationship with their GC contents, indicating that GC content might intensively shape codon usage in *A. gossypii*. Relationships of GC contents (estimated by GC3s: GC content at the third site of each codon) and codon usage bias (measured by Nc: effective number of codons which is a parameter measuring overall gene codon bias) on further analysis in all studies species show us that the higher GC3s is, the stronger correlation between GC3s and Nc is (Additional file 1B). For all studied orthologs in *A. gossypii*, GC3s is strongly correlated with Nc, but not strongly associated with tAI and CAI (codon adaptive index) (Additional file 1C), indicating that change of GC content might have a larger impact on gene Nc than on tAI and CAI values. As in Man and Piple [15], we obtained low correlations between tAI and Nc, and between CAI and Nc (Additional file 1C), which might be due to the dramatic change of GC content, thus Nc values in *A. gossypii*. Nevertheless, both correlations are still statistically significant, while tAI and CAI show a very strong positive correlation in this species (Additional file 1C). Because tAI values for orthologs among studied species were normalized to remove the impact caused by some factors at genome level including GC content, it would still be an accurate indicator for expression level, thus the importance of *A. gossypii *genes during evolution.

### Possible effect of protein size on gene translation efficiency

**Figure 4:**
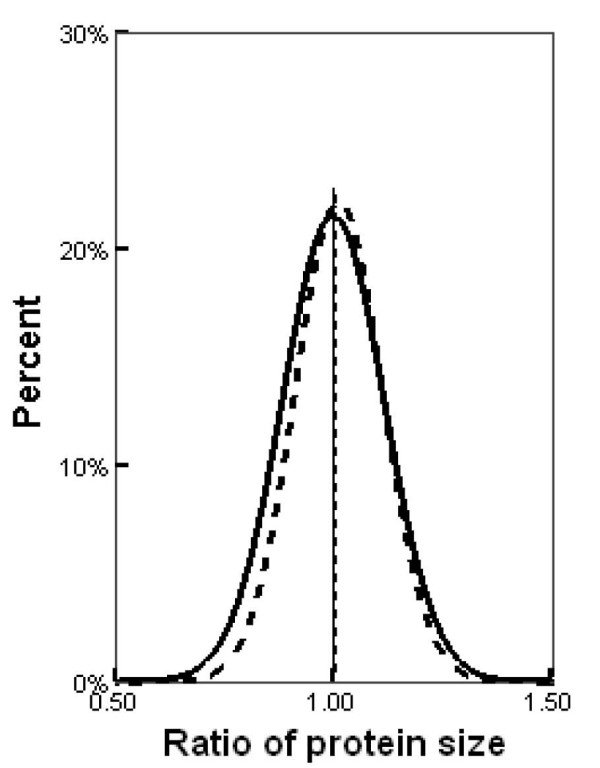
**Protein size distribution for Group I and Group II genes**. Protein size ratio for a gene was defined as the length of this gene in *A. gossypii *divided by the average length of its orthologous genes in nine yeast species. The solid and dashed lines represent the distributions of protein size ratio for Group I and Group II genes, respectively. Both distributions center around 1, indicating that there are no protein length differences between *A. gossypii *and studied yeast species for both Group I and Group II genes.

Natural selection biases synonymous codon usage to enhance accuracy and speed of protein synthesis in various organisms [11,12]. Differential gene translation efficiency reflects differences in functional requirements for proteins among organisms [14,15]. However, as previously reported, the negative correlation between translation efficiency and gene length indicates that translation efficiency could also be affected by protein size [14]. We tested if the different gene translation efficiencies observed between *A. gossypii *and yeast species could be caused by protein size deviation in different species. At first, we computed correlation coefficient of the tAI difference for each orthologs between *A. gossypii *and other nine yeasts and their protein size differences. To do this, for each orthologous gene, we calculated a protein size ratio, defined as the protein size of orthologous gene in *A. gossypii *(average protein size was used if two or more genes are in the same orthologous group) divided by the average protein size of orthologous genes in nine yeast species. Pearson correlation coefficient between tAI difference and protein size difference doesn't show significant association between the two (*r *= 0.035, *P*-value > 0.05). In addition, Distributions of protein size ratio for genes in Group I and II are shown in Figure 4. There are no significant differences in protein size between *A. gossypii *and yeast species for both Group I and Group II genes, indicating that our observations are not the results of protein size change of orthologous genes during evolution.

### Higher translation efficiency of cell cycle genes in *A. gossypii*

Comparison of translation efficiencies for orthologous genes should uncover regulation divergence, and thus the differential functional importance of those genes in diverged species [14,15]. Here by a comparative genomic approach, we identified two groups of orthologous genes showing differential translation efficiency between the filamentous fungus *A. gossypii *and its closely related yeast species. Genes in Group I have significantly higher translation efficiency in *A. gossypii *than those in yeasts. Functional analysis indicates that these genes are significantly enriched in cell cycle processes. Subsequent analysis of known pathways and experimentally studied genes in *A. gossypii *support such conclusion. Because enhanced translation efficiency indicates increased functional requirement for a gene, our results suggest that the roles of cell cycle genes might be substantially different between *A. gossypii *and yeasts. This suggestion is consistent with the observation that cell cycle processes, such as polar growth modes, cell cycle control and asynchronous nuclear division, are more complex in *A. gossypii *than those in yeast species [4,6,20,21]. Further experimental investigations for regulatory changes of cell cycle genes in *A. gossypii *could provide more insights into this conclusion.

For most pathogenic yeasts, transition from yeast form to filamentous form (pseudohyphal growth) is a key mark for their pathogenicity [1,22]. It has been reported that cell cycle genes play important roles in the morphological transition [1,23]. Micro-array experiment also shows that cell cycle genes are more up-regulated in *S. cerevisiae *filamentous form than are those in yeast form [1]. Our results indicate that cell cycle genes possess higher translational efficiency, and thus might be functionally more important, in filamentous fungus than those in yeast. Therefore, understanding species difference between filamentous fungus and yeasts could be helpful for illustrating mechanisms underlying filamentous growth of pathogenic yeasts.

### Higher translation efficiency for metabolic genes in yeasts

Genes in Group II have significantly lower translation efficiency in *A. gossypii *than those in yeasts. Functional enrichment analysis indicates that these genes are enriched for metabolic process, such as amino acid and steroid biosynthesis. This functional distribution might be associated with rapid cell growth in yeasts through fermentative life style. All yeast species studied can carry out fermentative growth under anaerobic or aerobic conditions, but *A. gossypii *can only conduct aerobic respiration [24]. The cell division rate is usually much faster for fermentative than for respiratory growth [25]. For eukaryotic organisms, cell size is an important control at check points for cell division [26,27]. In order to attain critical size, cells need to synthesize cellular components much more rapidly during fermentative than during respiratory growth. Therefore the metabolic processes should be in greater demand in yeasts than that in *A. gossypii*. The difference in metabolic rate could lead to differential translation efficiency for metabolic genes between *A. gossypii *and yeasts.

## Conclusion

Comparative studies revealed that more than 95% genes in multicellular organism *A. gossypii *share orthologs with unicellular organism *S. cerevisiae *[5]. It has been proposed that different growth modes between these species were controlled by a very similar gene set [4-6]. Our studies of orthologous genes among *A. gossypii *and several yeast species show higher translation efficiency of cell cycle genes in *A. gossypii*, which is consistent with more complicated cell cycle processes in this multicellular organism. On the other hand, our results are also consistent with the faster growth of unicellular yeasts, which leads to greater demands of gene products for metabolic processes. Emergence of multicellular organisms was one of the most profound developmental transitions in the history of life [28]. Our results in this study, and further investigation for the difference between *A. gossypii *and yeast species might also be important for understanding this process.

## Methods

### Species and sequence data

According to the phylogenetic tree in Fitzpatrick et al. (2006) [2], 12 genomes have been sequenced in the clade *Saccharomycotina*. Among these species, only *Ashbya gossypii *is classified as filamentous fungus. For the remaining 11 yeast species, nine were used in our study (*Saccharomyces cerevisiae, Saccharomyces paradoxus, Saccharomyces mikatae, Saccharomyces bayanus, Candida glabrata, Kluyveromyces polysporus, Kluyveromyces waltii, Saccharomyces kluyveri*, and *Kluyveromyces lactis*). *Saccharomyces kudriavzevii *and *Saccharomyces castellii *were not used due to their low sequencing coverage. The protein, coding and genomic sequences for recently sequenced *K. polysporus *are from Scannell *et al *2007 [29]. Sequences for other species were downloaded from Fungal Comparative Genomics database [30] and National Center for Biotechnology Information [31].

### Orthologous matrix

Using InParanoid [32] program, orthologs between every two species were identified. According to the results from InParanoid, we used MultiParanoid [33] to generate orthologous matrix among all studied species. Each row in the matrix corresponds to an orthlogous gene shared by all studied species and each column to a species. Duplicated genes detected by InParanoid in one species were classified into the same orthologous group. Only orthologous groups which contain at least one gene for each species were used in our study. As a result, an orthologous matrix with 3,151 rows (genes) and 10 columns (species) was generated.

### tRNA adaptation index (tAI)

First, we used tRNAscan-SE software (version 1.1) to predict copy numbers for all tRNA genes (tRNA gene pool) in each genome [34]. The tRNA pools among closely related species are conserved (See additional file 5 for the original data used to perform this analysis) [15]. For species with relatively low genome sequencing quality, we used tRNA pools from their closely related species, a same approach as in Ref 15: the predicted tRNA pool from *S. cerevisiae *was used to replace the ones in *S. byanus, S. paradoxus*, *S. mikatae *and the tRNA pool from *K. lactis *was used to replace the ones in *K. waltii *and *S. kluyveri*.

Second, given coding sequence for a gene, we used a modified tAI.R program [15] to estimate its tAI value in each species according to the tRNA pools (we kept all codons for methionines expect the start codons). When an orthologous group contains more than one gene in a species, the average tAI value of these genes was used for that species.

Finally, we normalized tAI value of each gene by median tAI value for all studied genes in the same species. For each orthologous group, we used the tAI values for nine yeast species to construct a normal distribution model, which was then used to estimate the probability of observing tAI value for the *A. gossypii *gene. Under 95% confidence level, we identified 333 (Group I) and 552 genes (Group II) for which orthologs from *A. gossypii *have significantly higher and lower tAI values than those from yeasts, respectively. The rest 2,266 genes without significantly different tAI values between *A. gossypii *and yeasts were classified in Group III.

### Functional enrichment analysis

GO (Gene Ontology) annotations of *S. cerevisiae *genes were used for our analysis. We identified functional enrichment of genes in each group (group I, II and III) by GO term finder [18]. The functional enrichments were determined at *P*-value < 0.05. Pathway information for *S. cerevisiae *was downloaded from KEGG database http://www.genome.jp/kegg/. All studied orthologous genes in *S. cerevisiae *were mapped onto these pathways. For each pathway, a chi-square test was used to calculate the probability of gene number distribution among each group.

## Authors' contributions

HJ and ZG designed the study. HJ and YZ analyzed the data. HJ, WW and ZG wrote the manuscript. JS prepared the figures and checked the manuscript. All authors read and approved the final manuscript.

## Supplementary Material

Additional file 1 **GC content and codon usage in *A. gossypii*.** Figure A shows the relationship between GC content and codon usage. X axis is the GC content for each species. Y axis is the correlation of whole genomic codon usage measured by RSCU (Relative Synonymous Codon Usage) between *A. gossypii *and other nine yeasts (Additional file 5). Figure B, using codonW http://codonw.sourceforge.net/, we calculated values for GC3s and Nc and obtained their correlation efficiencies in all studied species. X axis is the GC contents for each species. Y axis is the correlation coefficients between GC3s and Nc for each studied species. Figure C shows correlations among four indexes: tAI, GC3s, Nc and CAI in *A. gossypii*. All ribosomal protein gene sequences in *A. gossypii *were used as reference set for CAI calculation. Pearson correlation coefficients among these parameters were calculated. The lower triangle of table contains the Pearson's correlation coefficients and the upper triangle of table contains *P*-values Click here for file

Additional file 2 **tAI values for orthologous genes in Group III.** The figure shows the tAI values pattern for orthologous genes among all species in Group III (no significant tAI values between *A. gossypii *and other yeast species). The scale of tAI values is shown to the right of the figure. (Agos: *Ashbya gossypii*; Sces: *Saccharomyces cerevisiae; *Spar:*Saccharomyces paradoxus; *Smik:*Saccharomyces mikatae; *Sbay:*Saccharomyces bayanus; *Cgla:*Candida glabrata; *Kpol:*Kluyveromyces polysporus; *Kwal:*Kluyveromyces waltii*, Sklu: *Saccharomyces kluyveri; *Klac:*Kluyveromyces lactis*) Click here for file

Additional file 3 **The GO enrichment analyses for genes in Group I and II.** This table presents all functional categories defined by GO finder with *P*-value < 0.05 for genes in Group I and II. Click here for file

Additional file 4 **Additional KEGG pathways that show gene enrichment in Group I and II.** This table presents all pathways with *P*-value between 0.05 and 0.01. Click here for file

Additional file 5 **The correlation of codon usage and tRNA pools among species.** The lower triangle of the table contains the Spearman's correlation coefficients of codon usage in the whole genome. RSCU (Relative Synonymous Codon Usage) values were calculated for all codons in each species. Then correlations of RSCU values were calculated for each species pairs. The upper triangle of the table is the Spearman's correlation coefficients of tRNA gene number among all species. Click here for file
