# Supplementary figures and images for: Differential selection on gene translation efficiency between the filamentous fungus Ashbya gossypii and yeasts
Source: BMC Evol Biol. 2008 Dec 29;8:343. doi: 10.1186/1471-2148-8-343 (PMC2632675; doi:10.1186/1471-2148-8-343)

# Additional file 1

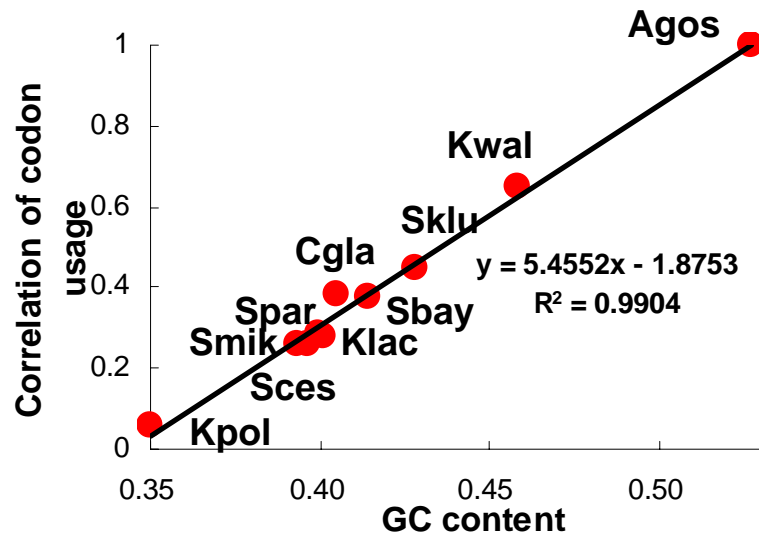

**A**

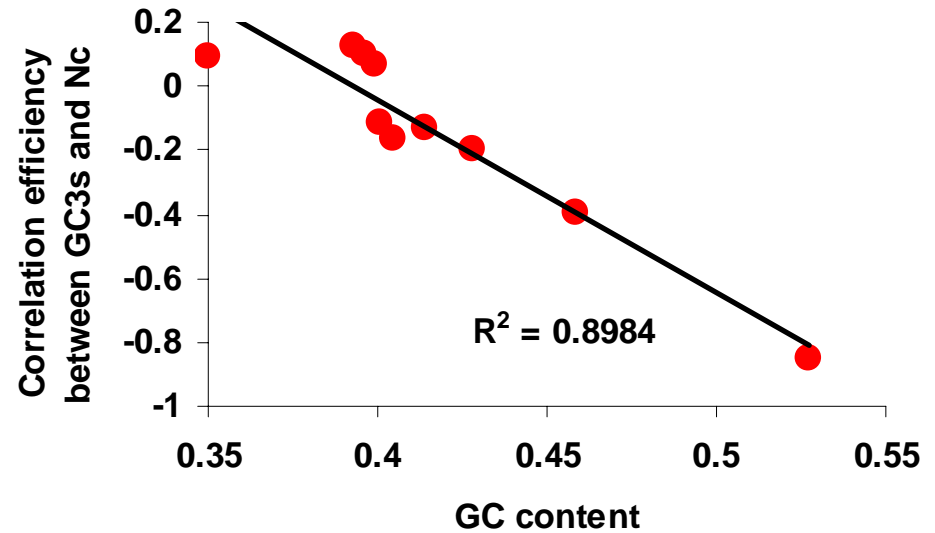

**B**

**C**

| Pearson Correlation | TAI   | CAI   | GC3s    | Nc       |
|---------------------|-------|-------|---------|----------|
| TAI                 |       | 0     | 1.6E-29 | 2.2E-161 |
| CAI                 | 0.90  |       | 2.2E-62 | 7.5E-285 |
| GC3s                | 0.16  | 0.24  |         | 0        |
| Nc                  | -0.38 | -0.49 | -0.85   |          |

Supplement: Additional file 1 — GC content and codon usage in A. gossypii. Figure A shows the relationship between GC content and codon usage. X axis is the GC content for each species. Y axis is the correlation of whole genomic codon usage measured by RSCU (Relative Synonymous Codon Usage) between A. gossypii and other nine yeasts (Additional file 5). Figure B, using codonW http://codonw.sourceforge.net/, we calculated values for GC3s and Nc and obtained their correlation efficiencies in all studied species. X axis is the GC contents for each species. Y axis is the correlation coefficients between GC3s and Nc for each studied species. Figure C shows correlations among four indexes: tAI, GC3s, Nc and CAI in A. gossypii. All ribosomal protein gene sequences in A. gossypii were used as reference set for CAI calculation. Pearson correlation coefficients among these parameters were calculated. The lower triangle of table contains the Pearson's correlation coefficients and the upper triangle of table contains P-values [file 1471-2148-8-343-S1.pdf]

## Additional file 2

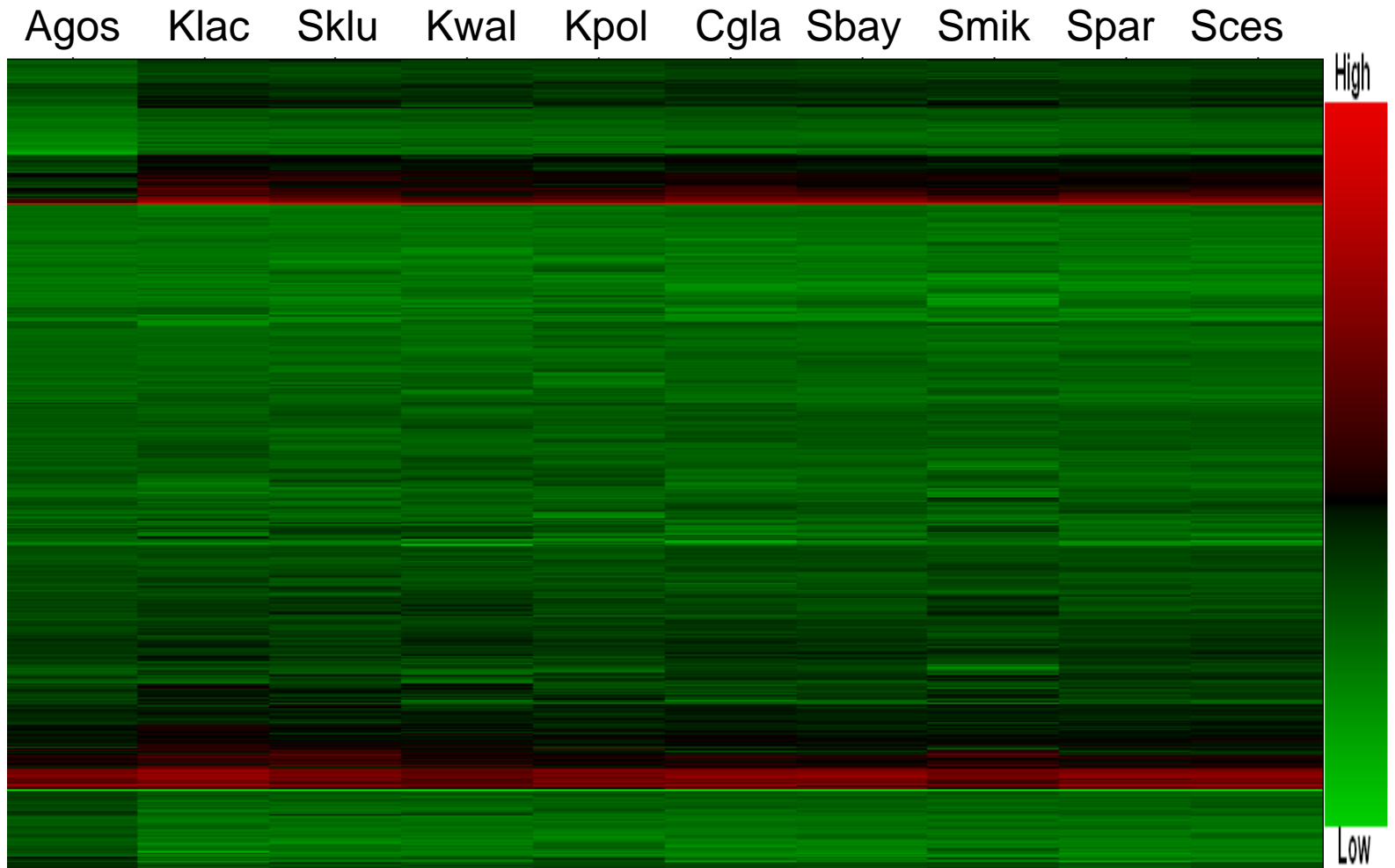

Supplement: Additional file 2 — tAI values for orthologous genes in Group III. The figure shows the tAI values pattern for orthologous genes among all species in Group III (no significant tAI values between A. gossypii and other yeast species). The scale of tAI values is shown to the right of the figure. (Agos: Ashbya gossypii; Sces: Saccharomyces cerevisiae; Spar:Saccharomyces paradoxus; Smik:Saccharomyces mikatae; Sbay:Saccharomyces bayanus; Cgla:Candida glabrata; Kpol:Kluyveromyces polysporus; Kwal:Kluyveromyces waltii, Sklu: Saccharomyces kluyveri; Klac:Kluyveromyces lactis) [file 1471-2148-8-343-S2.pdf]
